# Parents’ experience of perinatal post-mortem following stillbirth: A mixed methods study

**DOI:** 10.1371/journal.pone.0178475

**Published:** 2017-06-06

**Authors:** Jane Henderson, Maggie Redshaw

**Affiliations:** Policy Research Unit in Maternal Health and Care, National Perinatal Epidemiology Unit, Nuffield Department of Population Health, University of Oxford, Oxford, United Kingdom; University of Liverpool, UNITED KINGDOM

## Abstract

**Objectives:**

To analyse quantitative and qualitative data, to describe the experience of parents in relation to post-mortem following stillbirth, looking at offer and uptake of post-mortem, information-giving, the type of post-mortem carried out, receiving the results and any sociodemographic differences in care practices in relation to post-mortem.

**Design:**

Secondary analysis of a postal survey which included both open and closed questions allowing for a mixed methods study design.

**Population:**

Random sample of women who experienced a stillbirth in 2013.

**Methods:**

A sample of women who experienced a stillbirth in 2013 were selected by staff at the Office for National Statistics and sent a letter and information leaflet about the study followed by a postal questionnaire. The questionnaire included questions about pregnancy, labour and birth, the postnatal period, the time at which the baby died, and also asked about the post-mortem process.

**Results:**

Completed questionnaires were received from 477 women. Overall, 95% of women were asked for consent to a post-mortem, almost half prior to birth, and half by a consultant. The majority of women received written information and felt sufficiently informed, and agreed to a full post-mortem. A third of women had to wait longer than 12 weeks for the post-mortem result and this was the most common theme in the free text comments. Women also commented on the manner of being asked for consent, and wrote about issues related to communication and support. There were significant differences between sociodemographic groups in many of these factors.

**Conclusions:**

The inconsistencies in offer and consent to post-mortem following stillbirth suggest inequality in this area. The amount of time that many parents have to wait for post-mortem results is unacceptable and should be prioritised for improvement.

## Introduction

In the UK approximately one in 200 babies are stillborn after 24 weeks’ gestation, 4.16 stillbirths per 1000 total births [1]. This represents a continuation of a small and gradual decrease since 2004 but the UK still compares unfavourably with other high income countries [1].

Rates of perinatal post-mortem or perinatal autopsy have declined from 58% in 1993 to 42% in 2007, but this has increased slightly to 45% in 2014 [1–3]. The decline, which has been mirrored in other countries [4], is likely to be due to a number of different factors including the perceived usefulness of post-mortem, centralisation of pathology services, improved diagnostic imaging, adverse publicity around organ retention events in Bristol and Alder Hey hospital in the early 1990’s, and the subsequent introduction of a lengthier and more detailed consent form [3, 5]. This is unfortunate as there are many benefits to the parents and the extended family of perinatal post-mortem, principally to help determine the cause of death. A post-mortem has been shown to change the primary cause of death in between 9% and 76% of cases which compares favourably to placental histology and maternal blood tests [2, 6]. Other benefits of a post-mortem include the exclusion of possible contributory factors, confirming an antenatal diagnosis, identifying unsuspected conditions, and improved counselling for future pregnancies [1, 7]. In other research, parents also indicated that they felt it helped the grief process and hoped that it could benefit other parents [3].

A recommendation of the most recent Perinatal Mortality Surveillance Report for 2014 [1] was that all parents of stillborn babies should be offered a post-mortem which should preferably be conducted by a specialist pathologist, and that a placental histological examination should be carried out irrespective of consent for a post-mortem.

The manner in which the issue of post-mortem is discussed, the health professional raising the issue, and timing of the discussion all have a profound impact on the parents and the decision reached. It has been suggested that such discussions may in the past have been delegated to inexperienced junior staff [3]. However, several studies have refuted this, indicating that more senior obstetricians undertake these difficult conversations in between 63% and 95% of cases [2, 7, 8]. These studies also indicate that parents were generally satisfied with their decisions regarding post-mortem, findings were explained sufficiently and they had time to ask questions [2, 8, 9]; regret was greater amongst those who had declined post-mortem [2]. Of those parents who expressed dissatisfaction, the principle reasons related to the lack of established cause of death, to the use of medical jargon and the couple wanting more investigations [2, 8]. The conversation about post-mortem was usually either at the time of diagnosis or within the first few hours after the birth [2], and the issue was usually raised more than once although most parents in that study only recalled a single conversation [2]. Information was generally given verbally but most health professionals also provided written information [2].

Several studies also asked health professionals and parents about barriers to counselling parents regarding post-mortem [2, 3]. Health professionals indicated that a lack of rapport with the parents, a heavy workload, and emotional distress were important barriers. They also mentioned the negative press around organ retention, and religious or cultural barriers, but these were not significant issues for most parents. Important to parents was the possible necessity of the baby being transferred to a different hospital for post-mortem and the time required to obtain the results [2].

In these studies, reasons given by parents for declining a post-mortem included a feeling that the baby had gone through enough, that it would not help, that it would spoil the baby’s appearance, an assumption that antenatal ultrasound was sufficient, health professionals’ failure to give good explanations of the benefits of post-mortem, failure to discuss other options such as magnetic resonance imaging or a limited post-mortem, and a lack of understanding of religious and cultural concerns [3, 8, 10]. In general, there is an assumption by clinicians that relatives are unwilling to discuss autopsy. However, in a small study in the UK [11] 89% of relatives gave consent to autopsy.

With respect to maternity care, differences in the experience of various groups have been noted [12, 13]. However, there is a relative paucity of research on the experiences of parents of stillborn babies who are from minority, disadvantaged and other groups, including their experience of post-mortem. Many religious groups e.g. those of Jewish and Muslim faiths, believe that the body of a deceased person should be left intact and should be buried as soon as possible [10]. However, where there are over-riding concerns, such as the health and well-being of living people, these observances can be put aside or a compromise solution can be found [14].

This study aimed to analyse quantitative and qualitative data, to describe the experience of parents in relation to post-mortem, looking at offer and uptake of post-mortem, information-giving, the type of post-mortem carried out, receiving the results and any sociodemographic differences in care practices in relation to post-mortem.

## Methods

This study used secondary analysis of a postal survey, Listening to Parents, conducted in 2013 [15]. The survey was designed and conducted in collaboration with the charities Sands and Bliss. Different questionnaires were sent to mothers of babies who were stillborn and to mothers of babies who died in the neonatal period although this paper relates only to mothers of stillborns.

The Office for National Statistics (ONS) sampled women whose baby’s stillbirth had been registered between January and March 2013 or between June and August 2013. The women were sent information about the study, then the questionnaire by ONS at six to nine months after their baby’s stillbirth. A Freephone number to the project team was available for the duration of the study. A reminder letter together with a further questionnaire was sent to non-respondents four weeks later [15].

Questions were put to mothers about their maternity care, the postnatal period, and care at the time their baby died including a section about post-mortem. Differences between groups were tested using Chi-square statistics and analyses were conducted in Stata (version 13). A mixture of open and closed questions were used allowing for a mixed methods study design for subsequent research. These included questions about ‘Anything else you would like to say about your care around the time when you found out your baby had died’, ‘during the labour and stillbirth of your baby’, ‘in the maternity unit’ and at the end of the survey ‘anything else you would like to tell us about your care while you were pregnant or since your baby died’.

The open text responses were checked for material related to post-mortem. These were read and re-read independently in an iterative process coding material into over-arching themes and sub-themes as they arose using a thematic content analytic approach. Codes were further refined as the analysis progressed and differences in coding were resolved by discussion. Credibility and trustworthiness were tested using deviant case analysis and triangulation with quantitative data [16].

NHS Research Ethics approval for this study was obtained in July 2012 from National Research Ethics Service Committee South Central–Oxford A.

## Results

In total 1668 women who had a stillborn baby were invited to take part in the survey. Completed questionnaires were received from 477 women, a usable response rate of 30% (after removal of undelivered questionnaires from the denominator]. Of these, 419 women had a stillbirth where the baby had died before the onset of labour; 58 women had a stillbirth in which the baby died during labour. ONS provided limited information about women who had not responded to the survey allowing comparison with women who had responded. Non-responders were significantly more likely to have been born outside the UK, to be aged less than 30 and to live in a more deprived area [15]. This is similar to the patterns of response in surveys of women with live births [17, 18].

### Quantitative results

In this study raw percentages are used to describe the offer and uptake of post-mortem in different groups, along with details of the post-mortem such as timing of the discussion, information given, type of post-mortem carried out and perceived cause of death.

The proportions of women asked for consent to a post-mortem, when consent was requested and by whom, are shown by sociodemographic characteristics (Table 1).

**Table 1:** Proportions of women asked for consent to a post-mortem by sociodemographic characteristics (Excludes 56 babies for whom a post-mortem was not necessary (as indicated by the mother), principally because of congenital anomalies).

Asked for consent to post-mortem^1^	Yes		Asked before birth^1^		Asked by consultant	
	N	row %	N	row %	N	row %
Maternal age (years)						
16–19	18	81.8	6	35.3	8	44.4
20–24	55	94.8	15	29.4	26	47.3
25–29	87	95.6	34	43.6	40	46.0
30–34	139	97.2	68	56.7	72	51.8
35–39	70	94.6	35	59.3	40	57.1
40+	25	92.6	8	34.8	13	52.0
Total	394	94.9	166	47.7	199	50.5
Index of multiple deprivation						
1 (most deprived)	83	90.2	34	43.0	39	47.0
2	81	97.6	28	41.2	42	51.9
3	72	93.5	32	47.8	37	51.4
4	80	97.6	31	47.7	39	48.8
5	75	96.2	38	57.6	40	53.3
Total	391	94.9	163	47.3	197	50.4
Parity						
Primiparous	237	96.0	86	41.9	118	49.8
Multiparous	156	93.4	80	55.5	79	50.6
Total	393	94.9	166	47.7**	197	50.1
Ethnicity						
White	349	96.1	146	47.4	180	51.6
Mixed	5	100.0	2	40.0	2	40.0
Asian	26	96.3	12	54.6	7	26.9
Black	11	68.8	5	45.5	7	63.6
Other	2	66.7	1	50.0	1	50.0
Total	393	94.9**	166	47.7	197	50.1
Single mother						
No	362	95.3	154	48.3	183	50.6
Yes	33	91.7	12	38.7	16	48.5
Total	395	95.0	166	46.2	199	50.4
Left FT education aged <16 years						
No	327	95.1	140	49.0	162	49.5
Yes	60	93.8	23	40.4	33	55.0
Total	387	94.9	163	47.5	195	50.4
Gestational age at stillbirth (weeks)						
24–28	85	92.4	40	53.3	47	56.6
29–32	52	96.3	26	50.0	26	51.0
33–36	78	95.1	40	52.6	39	49.4
37 or more	165	95.9	51	36.4	77	46.4
Total	380	95.0	157	46.7	189	49.9

^1^ Denominator excludes women who had an intrapartum stillbirth

(**p<0.01)

The only significant difference was by ethnicity with Black women being substantially less likely to be asked for consent to post-mortem (White women 96%, Black women 69%). Teenagers were also less likely to be asked for consent but this difference was not statistically significant (p = 0.08). Consent was requested before birth in 37% of the deaths that occurred prior to labour and there was a significant difference by parity with primiparous women being asked for consent somewhat later than multiparous women (42% and 56% respectively). Duration of hospital stay following a stillbirth was slightly shorter than for women who had a live birth (1.82 and 2.17 days respectively). Therefore, there may have been little time for discussion of post-mortem. Consent was requested by a consultant in half of cases (51%). Women who had left full-time education aged less than 16 years were significantly more likely to be asked for consent by a midwife compared to women with more years of education (39% and 26% respectively).

Women were asked whether they received written information about the post-mortem, whether they felt informed enough to make a choice, and if they had enough time to make up their mind. While only two-thirds of women received written information, 85% and 81% respectively felt they were sufficiently informed and had enough time to decide. Teenagers and women aged 40 or more were significantly less likely to feel sufficiently informed (Table 2).

**Table 2:** Proportion of women who received written information, whether they felt sufficiently informed, and whether they had time to decide about post-mortem.

	Received written information about post-mortem		Felt sufficiently informed about post-mortem		Had sufficient time to make post-mortem decision	
	N	row %	N	row %	N	row %
Maternal age (years)						
16–19	12	57.1	16	80.0	16	76.2
20–24	39	75.0	49	87.5	47	83.9
25–29	54	62.1	76	85.4	73	81.1
30–34	96	72.2	120	86.3	114	81.4
35–39	48	69.6	59	84.3	58	82.9
40+	14	58.3	18	78.3	17	73.9
Total	263	68.1	338	85.1*	325	81.3
Index of multiple deprivation						
1 (most deprived)	44	57.1	68	82.9	61	75.3
2	53	67.1	61	76.3	59	73.8
3	54	73.0	63	85.1	61	80.3
4	56	70.9	73	89.0	74	90.2
5 (least deprived)	53	71.6	70	92.1	67	85.9
Total	260	67.9	335	85.0	322	81.1
Parity						
Primiparous	163	69.7	209	86.4	202	82.4
Multiparous	98	64.5	129	83.2	123	79.4
Total	261	67.6	338	85.1	325	81.3
Ethnicity						
White	236	69.2	296	84.8	285	81.2
Mixed	4	80.0	5	100.0	4	80.0
Asian	12	48.0	24	85.7	24	88.9
Black	7	53.8	11	84.6	10	71.4
Other	2	100.0	2	100.0	1	50.0
Total	261	67.6	338	85.1	324	81.2
Single mother						
No	242	68.6	315	85.4	302	81.4
Yes	21	60.0	25	83.3	25	80.6
Total	263	67.8	340	85.2	327	81.3
Left FT education aged <16 years						
No	223	69.3	285	85.6	279	82.8
Yes	37	61.7	50	83.3	43	74.1
Total	260	68.1	335	85.2	322	81.5

(* p<0.05)

Of the 416 women (87%) who were offered and asked to consent to post-mortem (excluding 56 whose babies did not need post-mortem (as indicated by the mother), mostly due to the presence of major congenital abnormalities), 81% consented to a full post-mortem, 7% to a partial post-mortem, 7% to an external examination only, and 5% to an examination of the placenta only (Table 3). Single mothers and women who had left full-time education before 16 years were significantly less likely to consent to a full post-mortem.

**Table 3:** Type of post-mortem by sociodemographic characteristics.

Type of post-mortem	Full post-mortem		Partial post-mortem		External exam only		Exam of placenta		Total	
	N	%	N	%	N	%	N	%	N	%
Maternal age (years)										
16–19	8	72.7	0	0.0	1	9.1	2	18.2	11	100
20–24	29	76.3	3	7.9	4	10.5	2	5.3	38	100
25–29	52	78.8	5	7.6	6	9.1	3	4.5	66	100
30–34	91	86.7	2	1.9	6	5.7	6	5.7	105	100
35–39	40	81.6	6	12.2	2	4.1	1	2.0	49	100
40+	11	68.8	3	18.8	2	12.5	0	0.0	16	100
Total	231	81.1	19	6.7	21	7.4	14	4.9	285	100
Index of multiple deprivation										
1 (most deprived)	38	74.5	4	7.8	4	7.8	5	9.8	51	100
2	48	82.8	3	5.2	4	6.9	3	5.2	58	100
3	40	71.4	8	14.3	7	12.5	1	1.8	56	100
4	50	86.2	2	3.4	3	5.2	3	5.2	58	100
5	52	88.1	2	3.4	3	5.1	2	3.4	59	100
Total	228	80.9	19	6.7	21	7.4	14	5.0	282	100
Parity										
Primiparous	150	80.6	10	5.4	16	8.6	10	5.4	186	100
Multiparous	80	82.5	8	8.2	5	5.2	4	4.1	97	100
Total	230	81.3	18	6.4	21	7.4	14	4.9	283	100
Ethnicity										
White	205	81.0	18	7.1	18	7.1	12	4.7	253	100
Mixed	5	100.0	0	0.0	0	0.0	0	0.0	5	100
Asian	11	78.6	0	0.0	1	7.1	2	14.3	14	100
Black	7	70.0	1	10	2	20	0	0.0	10	100
Other	1	100.0	0	0.0	0	0.0	0	0.0	1	100
Total	229	80.9	19	6.7	21	7.4	14	4.9	283	100
Single mother										
No	220	83.3	16	6.1	18	6.8	10	3.8	264	100
Yes	11	52.4	3	14.3	3	14.3	4	19.0	21	100
Total	231	81.1	19	6.7	21	7.4	14	4.9	285	100**
Left FT education aged <16 years										
No	199	82.6	15	6.2	20	8.3	7	2.9	241	100
Yes	29	74.4	4	10.3	1	2.6	5	12.8	39	100
Total	228	81.4	19	6.8	21	7.5	12	4.3	280	100*

* p<0.05,

** p<0.01

Of the 110 women who declined any form of post-mortem, the most common reason given (74%) was that they didn’t want their baby’s body examined, 38% indicated they already knew why their baby had died, and 26% thought that it would not provide an answer. Only 11% of women indicated that it was against their beliefs, but this reason was given significantly more often among women living in the most deprived areas and those of Mixed or Asian ethnicity (Table 4).

**Table 4:** Main reasons given for declining post-mortem by socioeconomic characteristics.

	Didn't want body examined		No need, knew why baby died		Wouldn't give an answer		Against beliefs	
	N	row %	N	row %	N	row %	N	row %
Maternal age (years)								
16–19	12	92.3	2	15.4	2	15.4	2	15.4
20–24	21	63.6	16	48.5	8	24.2	5	15.2
25–29	36	80.0	17	37.8	10	22.2	7	15.6
30–34	44	75.9	24	41.4	15	25.9	3	5.2
35–39	21	63.6	13	39.4	10	30.3	3	9.1
40+	9	81.8	1	9.1	5	45.5	1	9.1
Total	143	74.1	73	37.8	50	25.9	21	10.9
Index of multiple deprivation								
1 (most deprived)	39	72.2	15	27.8	8	14.8	12	22.2
2	31	86.1	11	30.6	11	30.6	5	13.9
3	24	68.6	19	54.3	8	22.9	1	2.9
4	25	64.1	20	51.3	12	30.8	3	7.7
5	24	82.8	8	27.6	11	37.9	0	0.0
Total	143	74.1	73	37.8*	50	25.9	21	10.9**
Parity								
Primiparous	77	79.4	31	32.0	24	24.7	13	13.4
Multiparous	68	69.4	42	42.9	26	26.5	8	8.2
Total	145	74.4	73	37.4	50	25.6	21	10.8
Ethnicity								
White	127	77.0	62	37.6	45	27.3	9	5.5
Mixed	12	63.2	6	31.6	4	21.1	10	52.6
Asian	5	55.6	4	44.4	1	11.1	2	22.2
Black	1	100.0	0	0.0	0	0.0	0	0.0
Total	145	74.7	72	37.1	50	25.8	21	10.8**
Single mother								
No	127	74.3	64	37.4	44	25.7	18	10.5
Yes	18	75.0	9	37.5	6	25.0	3	12.5
Total	145	74.4	73	37.4	50	25.6	21	10.8
Left FT education aged <16 years								
No	114	75.0	60	39.5	42	27.6	16	10.5
Yes	26	72.2	11	30.6	7	19.4	2	5.6
Total	140	74.5	71	37.8	49	26.1	18	9.6

* p<0.05,

**p<0.01

Overall, 5% of women received the results of the post-mortem within 4 weeks, 31% within 5–8 weeks, 34% within 9–12 weeks, and 30% waited longer than 12 weeks (Table 5). There were no significant differences by sociodemographic characteristics but a longer wait for results was slightly (but not statistically significantly) associated with placental problems given as a cause of death. The vast majority of women received the results in a meeting with a consultant obstetrician although women of Mixed or Other ethnicity, and those with less education were significantly less likely than other women to receive the results from the consultant (Table 6). Overall, a bereavement midwife conveyed the results or was present in 10% of cases. Their support was greatly appreciated as indicated by the qualitative results below. A few women (10 in total) received the post-mortem result by letter or email.

**Table 5:** How long women had to wait for post-mortem results by sociodemographic factors.

Time to post-mortem results	4 weeks or less		5–8 weeks		9–12 weeks		>12 weeks		Total	
	N	%	N	%	N	%	N	%	N	%
Maternal age (years)										
16–19	2	18.2	2	18.2	3	27.3	4	36.4	11	100
20–24	3	8.1	12	32.4	11	29.7	11	29.7	37	100
25–29	2	3.2	22	34.9	19	30.2	20	31.7	63	100
30–34	4	3.8	27	26.0	43	41.3	30	28.8	104	100
35–39	1	2.0	16	32.7	16	32.7	16	32.7	49	100
40+	1	6.3	7	43.8	4	25.0	4	25.0	16	100
Total	13	4.6	86	30.7	96	34.3	85	30.4	280	100
Index of multiple deprivation										
1 (most deprived)	0	0.0	18	37.5	15	31.3	15	31.3	48	100
2	4	6.9	13	22.4	21	36.2	20	34.5	58	100
3	3	5.5	20	36.4	17	30.9	15	27.3	55	100
4	4	6.9	16	27.6	20	34.5	18	31.0	58	100
5 (least deprived)	2	3.4	18	31.0	22	37.9	16	27.6	58	100
Total	13	4.7	85	30.7	95	34.3	84	30.3	277	100
Parity										
Primiparous	7	3.9	62	34.4	57	31.7	54	30.0	180	100
Multiparous	6	6.1	23	23.5	39	39.8	30	30.6	98	100
Total	13	4.7	85	30.6	96	34.5	84	30.2	278	100
Ethnicity										
White	11	4.4	73	29.2	87	34.8	79	31.6	250	100
Mixed	0	0.0	2	40.0	3	60.0	0	0.0	5	100
Asian	1	7.7	5	38.5	2	15.4	5	38.5	13	100
Black	1	11.1	4	44.4	3	33.3	1	11.1	9	100
Other	0	0.0	0	0.0	1	100.0	0	0.0	1	100
Total	13	4.7	84	30.2	96	34.5	85	30.6	278	100
Single mother										
No	10	3.9	80	30.9	90	34.7	79	30.5	259	100
Yes	3	14.3	6	28.6	6	28.6	6	28.6	21	100
Total	13	4.6	86	30.7	96	34.3	85	30.4	280	100
Left FT education aged <16 years										
No	12	5.0	73	30.7	80	33.6	73	30.7	238	100
Yes	1	2.6	10	26.3	16	42.1	11	28.9	38	100
Total	13	4.7	83	30.1	96	34.8	84	30.4	276	100

**Table 6:** How post-mortem results were conveyed, by consultant and/or by bereavement midwife^1^ (as indicated by the mother).

	Consultant^1^		Bereavement midwife^1^	
	N	%	N	%
Maternal age (years)				
16–19	10	90.9	1	9.1
20–24	37	94.9	4	10.3
25–29	56	84.8	11	16.7
30–34	97	94.2	8	7.8
35–39	46	92.0	5	10.0
40+	15	93.8	0	0.0
Total	261	91.6	29	10.2
Index of multiple deprivation				
1 (most deprived)	47	92.2	5	9.8
2	53	91.4	5	8.6
3	48	87.3	8	14.5
4	54	91.5	9	15.3
5	56	94.9	2	3.4
Total	258	91.5	29	10.3
Parity				
Primiparous	171	92.4	21	11.4
Multiparous	88	89.8	7	7.1
Total	259	91.5	28	9.9
Ethnicity				
White	234	92.9	26	10.3
Mixed	3	60.0	1	20.0
Asian	12	85.7	1	7.1
Black	9	90.0	0	0.0
Other	1	50.0	0	0.0
Single mother				
No	242	92.0	27	10.3
Yes	19	86.4	2	9.1
Total	261	91.6	29	10.2
Left FT education aged <16 years				
No	226	93.4	24	9.9
Yes	31	79.5	4	10.3
Total	257	91.5*	28	10.0

^1^ Not exclusive categories, both consultant and bereavement midwife may have been present.

(* p<0.05)

Maternal understanding of the cause of the stillbirth is shown in Table 7. Where a post-mortem was carried out, 29% of stillbirths were unexplained, compared to 37% where no post-mortem was done. There were significant differences in perceived cause of death by parity and ethnicity with primiparous women being more likely to report that the stillbirth was due to growth restriction and multiparous women that it was due to a congenital abnormality. White women were significantly more likely to report that death was due to placental or cord problems compared to women from other ethnicities, although maternal perception may not be an accurate reflection of reality.

**Table 7:** Parental understanding of cause of death.

Cause of death	Congenital abnormality		Growth restriction		Preterm		Placental/cord problem		Unexplained	
	N	row %	N	row %	N	row %	N	row %	N	row %
Maternal age (years)										
16–19	2	8.7	2	8.7	0	0.0	12	52.2	8	34.8
20–24	9	13.0	12	17.4	3	4.3	33	47.8	19	27.5
25–29	11	10.9	15	14.9	2	2.0	50	49.5	37	36.6
30–34	24	15.2	22	13.9	5	3.2	67	42.4	56	35.4
35–39	11	13.8	10	12.5	0	0.0	36	45.0	23	28.7
40+	2	8.0	1	4.0	1	4.0	7	28.0	11	44.0
Total	59	12.9	62	13.6	11	2.4	205	45.0	154	33.8
Index of multiple deprivation										
1 (most deprived)	16	15.7	14	13.7	4	3.9	43	42.2	34	33.3
2	12	13.2	12	13.2	1	1.1	38	41.8	33	36.3
3	9	10.2	14	15.9	2	2.3	42	47.7	29	33.0
4	12	13.0	10	10.9	2	2.2	43	46.7	25	27.2
5	9	11.3	11	13.8	2	2.5	37	46.3	32	40.0
Total	58	12.8	61	13.5	11	2.4	203	44.8	153	33.8
Parity										
Primiparous	27	10.1	45	16.8	4	1.5	129	48.1	91	34.0
Multiparous	32	17.0	17	9.0	7	3.7	77	41.0	62	33.0
Total	59	12.9*	62	13.6*	11	2.4	206	45.2	153	33.6
Ethnicity										
White	46	11.6	56	14.1	8	2.0	191	48.1	129	32.5
Mixed	1	16.7	0	0.0	0	0.0	0	0.0	4	66.7
Asian	7	21.9	4	12.5	2	6.3	9	28.1	13	40.6
Black	3	16.7	2	11.1	1	5.6	5	27.8	8	44.4
Other	1	50.0	0	0.0	0	0.0	0	0.0	0	0.0
Total	58	12.7	62	13.6	11	2.4	205	45.1**	154	33.8
Single mother										
No	54	13.0	57	13.7	10	2.4	189	45.4	142	34.1
Yes	5	11.9	5	11.9	1	2.4	18	42.9	12	28.6
Total	59	12.9	62	13.5	11	2.4	207	45.2	154	33.6
Left FT education aged <16 years										
No	52	13.8	53	14.1	11	2.9	163	43.4	128	34.0
Yes	5	6.9	9	12.5	0	0.0	39	54.2	23	31.9
Total	57	12.7	62	13.8	11	2.5	202	45.1	151	33.7

* p<0.05,

** p<0.01

### Qualitative results

Three main themes were identified in the qualitative analysis: ‘Consent for post-mortem’, ‘Getting the results’ and ‘Communication and support’ (Table 8). Each theme and related sub-themes is illustrated and discussed. Women are identified in the quotes only by a number as further details may compromise anonymity. Only the women were surveyed, however, ‘we’ and ‘us’ were commonly used in writing about the couple’s experience of the post-mortem process.

**Table 8:** Themes identified in qualitative analysis of open text responses.

*Themes*	*Sub-themes*
**Consent for post-mortem**	Timing of the question
	The way it was asked
**Getting the results**	The time it took
	The impact of delay
	Unanswered questions
**Communication and support**	Taking time to listen and explain
	Following up

### Consent for post-mortem

The context of consent and the consent process are complex socially and psychologically for both parents whose baby has just died and the staff caring for them. Two sub-themes were evident in the open text responses: ‘Timing of the question’ and ‘The way it was asked’.

#### Timing of the question

Interpersonal skills and experience are likely to contribute to the way parents are asked. Some, though not all, felt the pressure to have a post-mortem, felt rushed and that they were asked too soon:

‘A Doctor came in to talk to us about post-mortem half an hour after the birth—this was too early to take in the information—we had to ask her to come back later.’

[11588]

*‘Short time after the delivery I was left alone*, *not given a ring bell if I needed*. *I was given*, *I think*, *not too sure*, *anaesthetics for stitches which made me very vulnerable and interfered at that time and asked to fill in post-mortem application which was left away from me*.’

[10403]

#### The way it was asked

Other parents emphasised the way the request for consent was managed, reporting a lack of sensitivity on the part of the clinical staff and a need to get this task done:

*‘After my baby was born when we wanted to spend longer with our baby the Midwife rushed us by trying to go through post-mortem consent form… we feel robbed of memories we could have made*, *and time with our baby*. *Our wishes were seemingly unimportant*.’

[11207]

‘We had a registrar in the room as a witness while we were signing consent on post-mortem. She was very insensitive—yawning, tapping pen, rolling eyes and just generally being inconsiderate. I feel a Midwife should have witnessed as we were comfortable with them.’

[11734]

One mother contrasted this with what had gone before:

‘Saw two Doctors, [name] was excellent, one female doctor was trying to push us into a post-mortem several times, she seemed to be "putting on" sympathy than genuine empathy with us. Felt over-pressured to have a post-mortem, they were very quick to send me away before my baby had died but wanted to examine him after he died. Too late.’

[10134]

While some parents clearly felt pressed and hurried into the process, another couple had to make a considerable effort to get a post-mortem and then did not feel confident about the health professional obtaining consent:

*‘After giving birth, there was no Doctor available to discuss and sign the post-mortem forms. We decided to go home after waiting several hours and come back the next day after being called to say a Doctor was available. Once again we were left waiting and the Doctor that did see us, had no idea, didn't explain anything and seemed like she had never seen the forms before’*.

[10667]

### Getting the results

Most of the sub-themes that were identified were closely tied up with the issue of timing: the time estimated to get results and delays; what parents felt they had to do in order to get the post-mortem findings and the effect on parental wellbeing of waiting to hear.

#### The time it took

By far the most common theme was the length of time it took to get the post-mortem results, with some parents contrasting the process with other aspects of care:

‘… if we had been told from the beginning 20 weeks instead of 6–8 weeks we may have made a different decision….’

[10667]

‘I cannot fault the care I received—it was human, compassionate, personal and touching. The only difficulty was in waiting for the post-mortem as it took so long. We were booked in at 12 weeks after delivery and we turned up just to find that the post-mortem was not ready. We were understanding, as it was nobody's fault. But five months is a long time to hold on for answers before you can start to move forward.’

[11158]

‘My only negative experience with care after my daughter’s stillbirth was in the follow up appointment with the consultant to receive post-mortem results. I had to phone on several occasions as the consultant’s secretary said he had not had time to read the report yet. This went on for approx 4 weeks. It was very distressing to have other appointments at the hospital and know the results were just sat on someone's desk there.’

[11291]

‘..the length of time that the post-mortem took was far too long. We had buried our baby, but we still had the results of the post-mortem outstanding and hanging over us. All these issues proved very disappointing after receiving such good care whilst in hospital.’

[11008]

There were many other responses of a similar nature; one mother was still waiting for the results at the time of completing the survey 10 months after the birth.

#### Chasing the findings

A related theme was having to chase up the results often involving multiple calls to administrators and health professionals. Poor communication appeared to underpin some parents’ experiences:

*‘Every time we spoke to the hospital whilst waiting for the post-mortem results a different time frame was given to us*, *varying from 6–8 weeks*, *then 8–12 weeks and finally 12–18 weeks*. *The phone calls were put through to a different department and different answers were given each time*. *It took a few weeks before we were told which consultant is dealing with our case and how to contact the consultant*…. *a summary of the post-mortem result was posted to a previous address resulting in further delay of two months before we received the letter*.*’*

[10710]

‘…. each time I called I had to explain the situation to a different person. The medical advice was excellent when we got it but was a little let down by poor administration. This is only important because in that situation, the "patient" is hanging on the every word of the doctors and so it is essential that time frames and next steps are clear and that the goal posts don't change.’

[10770]

‘The process of obtaining an explanation for my baby’s death was difficult…. I lost count of how many times I had to chase the results. The post-mortem was carried out… and it took 19 weeks to get someone to explain the results to me.’

[11136]

‘Getting a fixed appointment for the post-mortem results took lots of phone calls and effort. It was then treated as a 'one-off' appointment, and as if we were being done a favour.’

[11646]

#### The impact of delay

Parents described the distress experienced in the interval before receiving the post-mortem findings: *‘the bureaucracy it took get to that stage was very upsetting*,*’* and the delays led to parents *‘holding on’*, ‘*waiting to move forward’* and feeling *‘the grieving process was set back’*. For many it was *‘one of the hardest parts*.*’* They clearly felt held back, not able to move on and obliged to wait or participate in chasing results:

‘Waiting so long meant moving forward with our lives was very hard as we were continuously worrying about the results. It was a very stressful time.’

[10667]

*‘The post-mortem was the most traumatic as we were told by the consultant that it would take 2 weeks. 14 weeks later we finally got the results. So in between that time I felt like I couldn't move on as I was constantly waiting’*.

[10253]

‘After the labour I was told I would receive an appointment with the consultant after 12 weeks. On the 12th week I had heard nothing and chased the hospital. I had a month of distress, and promises before I received my consultant’s appointment.’

[11358]

Some women felt they were no longer valued and that their needs were not recognised in the context of feeding back the post-mortem findings following stillbirth:

*‘Nobody from the hospital contacted me*. *I had to constantly ring to find if my baby had gone / finished post-mortem*…. *I felt the hospital abandoned* me.’

[10795]

‘I felt 'left out'. I had to make all the calls to find out when my son would be back from the post-mortem and when or if I could see him again.’

[10597]

‘I was sent a letter to visit the hospital to discuss the death of my baby, weeks after. Do they not understand PTSD and how leaving the house is difficult, how could I travel miles when I am still to this day suffering and on medication with panic attacks, suicidal thoughts. Feel so let down.’

[11342]

#### Unanswered questions

The theme of ‘unanswered questions’ was also identified in the responses of some women, particularly in the context of inconclusive results from the post-mortem:

‘Although the post-mortem was inconclusive, it did mention that the placenta showed signs of "Immaturity" in parts. Why can scans not be done to look specifically at the placenta development so that it can be avoided in the future?’

[10185]

‘When the consultant obstetrician met with us to discuss the post-mortem results he noted that our baby had been consistently small, but neither he nor his colleagues picked up on this and it left me wondering what was the point of offering all those extra appointments if no-one was being mindful of what they were recording.’

[11709]

This negative theme arose in relation to unsympathetic or even unkind treatment. Although this was mentioned only occasionally, clearly it should never occur.

‘*We only had one appointment with the consultant in which she 'threw' the post-mortem results at us and said "this is what you've been pestering for"*. *We were only given a short time with her and seeing as the post-mortem brought up 3 major issues we needed far longer to be able to digest all the information*.*’*

[10708]

‘Our post-mortem meeting with a consultant was unsatisfactory. The meeting started about 1/2 an hour late (I mean how can you allow yourself to be late for a meeting like that—you should plan ahead). The meeting was rushed, we felt dismissed with the consultant on his feet and ready to go before I got out my list of questions.’

[11521]

A few parents felt they had to work hard to understand the implications of the post-mortem results, needing further opportunities to ask questions of the clinicians:

‘Our post-mortem results indicated that the cause of our baby's death was Group B Strep infection (before labour, before waters breaking). The consultant was not familiar with such cause of death and, as no preparation had been done for the meeting, could only offer to find out some more information for a second meeting. We had a second and a third meeting with the consultant and felt we had to ask a lot of questions in order to get some reasonable answers (although some questions were never answered).’

[10203]

‘I could not take in the full information in the first appointment, once I had digested the information I had lots of questions which I needed to ask before I could start to rebuild my life. The consultant was horrible as I requested a second appointment.’

[11358]

### Communication and support

Several sub-themes centred on support and the provision of helpful, timely information and a need for further conversations and follow-up.

#### Taking time to listen and explain

Having a known midwife with whom to discuss the post-mortem, having the support of a bereavement midwife and health professionals taking the time to listen and talk over the findings made a difference:

‘The Midwife who delivered our baby attended the post-mortem appointment with us and will remain firmly dear to us in the future.’

[11510]

‘… once in the room with the Consultant he was very caring, interested in our current emotional state, and answered all our questions.’

[11646]

Communication was an important issue and the assumptions made upset some women. A lack of clarity about the appointment was evident:

‘The letter from the hospital inviting us to an appointment with the consultant after the stillbirth of our baby started with 'I am writing to offer you a follow-up appointment to discuss care in future pregnancies'. I found this terminology quite distressing as, at this time (around 3 weeks after my stillbirth), I wanted to understand why my baby had died and the thought of future pregnancies were not appropriate at that time. At this first meeting the consultant had not prepared ahead (we were told he was reading our notes when we arrived).’

[10203]

‘After 12 weeks I rang the hospital…. The women’s unit receptionists were off hand. It was only after we spoke to our GP did we get an appointment to see a consultant. The consultant told us when we had that appointment that our baby's post-mortem results had been with them for 4 weeks. As we had gone there just for a chat about trying for another baby, we didn't take in the details. No copy was offered.’

[11391]

‘…we were left feeling that our child's post-mortem report was at the bottom of the consultant’s in-tray and low priority—I believe it should be top priority! I also found out the blood results, and the reasons why my baby died via a letter I was cc'd into. I found this totally unacceptable.’

[11358]

Some women were distressed about the way in which the post-mortem results were conveyed or indeed the absence of results:

‘When we did see the consultant for the results she wasn't very sympathetic or understanding. I wanted to be reassured about what had happened and the future but instead came away feeling more worried and concerned.’

[10667]

‘Doctor wasn’t overly compassionate surrounding post-mortem information, quite blunt… Saw my consultant twice, once to confirm death and then to give me post-mortem results.’

[11093]

‘When I went to see the consultant about the post-mortem results she didn't know why I was there and had misplaced the results.’

[11177]

#### Following up

Another common theme was the lack of follow-up for parents after post-mortem. The difficulty of taking in information when in a highly stressed situation is well-known [19] and many parents found that further questions arose later.

*‘Our consultant left his job 2 weeks after he gave us the post-mortem results. He did not seem to care, gave us no support, just said it was one of those things… I've had no follow up appointments or investigations…. Feel let down’*.

[10618]

‘We weren't offered a follow-up appointment to discuss anything we may have thought of after receiving the results. This lack of care afterwards I found to be disgusting, and very detrimental to our emotional and mental wellbeing.’

[10708]

### Correspondence between quantitative and qualitative results

The use of mixed methods illustrates both the experience and feelings of those most involved. The women’s own words and the qualitative analysis reflect specific aspects of care about which they were critical. Differences in timing and parental concern about this were evident in the two types of data. Nearly half of women had been first asked about a post-mortem before birth and a large majority (81%) considered that they had enough time to decide. There was considerable individual variation in timing of request and response as was reflected in the quantitative and qualitative data, with some women feeling that they had been asked too soon. The variable wait for post-mortem results documented in the quantitative data coloured their experience as described in the qualitative material. Almost a third of women had to wait for longer than 12 weeks for the results and this is consistent with the ‘getting the results’ theme and sub-themes about ‘the time it took’ and ‘chasing’ in obtaining the findings being the most common complaint in the free-text responses. However, there was no association between time waiting for results and overall satisfaction with care after the baby had died.

## Discussion

Using a mixed methods approach this study has found that some aspects of the post-mortem are of particular concern to women who had experienced a stillbirth and are of direct relevance to those providing care. The manner of the health professional who asks about post-mortem and gives information and the way in which this is done matters to women, their partners and their care providers enormously. This concurs with recent smaller scale studies such as those carried out in one large tertiary centre in Ireland [20, 21].

Women of Black ethnicity were significantly less likely to be asked for consent to a post-mortem, teenagers and women aged over 35 years were significantly less likely to feel sufficiently informed regarding post-mortem, marginalised women, especially those with less education and without a partner, were significantly less likely to consent to a full post-mortem, and BME women and those with less education were significantly less likely to receive the results in a meeting with the consultant. These differences may relate partly to communication problems between health professionals and women from ethnic minority groups, from more deprived background and with less education. There may also be assumptions about religious and cultural observances that may preclude post-mortem. However, all couples suffering a stillbirth should be offered a perinatal post-mortem with appropriate counselling about the different forms it can take, and if burial needs to done within a specified period than it should be possible to expedite the procedure.

Overall, nearly half of respondents agreed to a full post-mortem (48% of all respondents, 58% of those asked for consent) which is a higher proportion than the national average of 45% [1] reflecting the nature of this sample. However, this was similar to the rates reported in an earlier UK study [2] in which 61.5% parents responding agreed to a post-mortem, but markedly lower than a Swedish study in 2004–5 in which 83% of women agreed to a post-mortem [9].

In this survey, as with other surveys of women who had a live baby [17, 18], there was significant under-representation of women who were born outside the UK, aged less than 30 and living in a more deprived area. The response rate of 30% is a limitation which makes generalisation difficult. However, it should be held in mind that a wide range of information was collected from those who did respond following such a life-changing experience, using a method which avoided the potential biases of online surveys. Although the Listening to Parents study is the largest study of women’s experience of stillbirths conducted, the numbers of women in the sub-groups was too small to achieve statistical significance in some of the sub-group analyses.

In general, the findings of this study are consistent with the literature in respect of the timing of the post-mortem discussion, the proportion of women offered a post-mortem, verbal and written information received, and who the discussion was with, although there were some differences compared to an online study [2]. In that study, 71% of women said that the discussion was with a consultant whereas the comparable figure in this study was 51%, and only 51% of women in the online study said that they received written information compared to 68% in this study. The reasons given for declining a post-mortem were also broadly consistent with the literature [3, 8, 10], with mothers indicating that they did not want their baby’s body examined, a post-mortem was not required as they already knew why their baby had died, or they did not think it would provide an answer. In the online study cited above, 65% of midwives and 56% of obstetricians, but only 3% of parents, considered religion and culture to be significant barriers to consent for post-mortem. In this study 11% of women gave ‘Against their beliefs’ as a reason for declining a post-mortem.

In the online study cited above, a quarter of midwives and 12% of obstetricians received no training in perinatal post-mortem, and a further 33% of midwives and 11% of obstetricians were dissatisfied with the training received [2]. All health professionals who responded to the online survey had incomplete knowledge of the post-mortem procedure and a third undervalued it [2] reflecting considerable scope for improved training in this area. A systematic review of interventions to support parents’ decisions about autopsy after stillbirth found no randomised controlled trials and concluded that parents had to rely on the ad-hoc knowledge and experience of those involved at the time [22].

In this study, almost a third of women had to wait longer than 12 weeks for the results of the post-mortem. This was the most common theme in the qualitative analysis and is consistent with other research [9]. However, the time needed for post-mortem results to come back was a reason for declining post-mortem in only 8% of cases and clearly such a long wait was not anticipated for most women. In the qualitative analysis, the feeling of being ‘in limbo’ whilst waiting for the results was exacerbated by incorrect information about how long the post-mortem would take, leaving the parents to ‘chase’ the results, and sometimes by unkindness and inefficiency in the way their concerns about delays were handled. NHS England quality and audit standards indicate that 60% of post-mortem reports should be issued within 42 days of examination and 90% with 56 days [23]. These standards were clearly not being met at the time of this survey in 2013. Anecdotal evidence suggests that most families are not told clearly how long it will take for post-mortem results to come back (personal communication: Charlotte Bevan). Delays may be caused by the time taken to organise an appointment with the consultant to communicate the results to the parents rather than the post-mortem process itself, although that can also be time consuming if chromosome analysis and placental examination are necessary [9].

## Conclusions

In this study perinatal post-mortem was conducted more frequently than the national average but there were inconsistencies in who it was offered to and in who consented to a full post-mortem. Women in more disadvantaged positions in society were less likely to be offered, or consent to, a post-mortem, and less likely to receive the results in a meeting with a consultant. The timing and sensitivity with which women were initially approached was commented on as was the time they had to wait for the post-mortem results. This was unacceptably long in many cases and women described how this impacted on the emotional wellbeing of themselves and their partner.

### Implications for practice

This study has implications for the training of midwives and obstetricians. All women should be offered post-mortem following a stillbirth and the discussions of possible benefits should be carried out sensitively by a consultant, a midwife known to the woman or a specialist bereavement midwife. Religious and cultural concerns are not reasons for failing to raise the topic. Results should be fed back to parents in a face to face discussion within the timeframe indicated by NHS England. Follow-up visits should be available to all parents who need or want them.

### Implications for research

There are no clear reasons for many of the sociodemographic differences found here. In particular, it is unclear why Black women were less likely to be asked for consent to a post-mortem. This is an area requiring further study. There is also a need for research into interventions to support parents in making decisions around post-mortem after stillbirth, as highlighted by an earlier systematic review [22].
